# Epic Allies, a Gamified Mobile Phone App to Improve Engagement in Care, Antiretroviral Uptake, and Adherence Among Young Men Who Have Sex With Men and Young Transgender Women Who Have Sex With Men: Protocol for a Randomized Controlled Trial

**DOI:** 10.2196/resprot.8811

**Published:** 2018-04-05

**Authors:** Sara LeGrand, Kathryn E Muessig, Alyssa Platt, Karina Soni, Joseph R Egger, Nkechinyere Nwoko, Tobias McNulty, Lisa B Hightow-Weidman

**Affiliations:** ^1^ Center for Health Policy and Inequalities Research Duke Global Health Institute Duke University Durham, NC United States; ^2^ Department of Health Behavior Gillings School of Global Public Health University of North Carolina at Chapel Hill Chapel Hill, NC United States; ^3^ Duke Global Health Institute Duke University Durham, NC United States; ^4^ Division of Infectious Diseases School of Medicine University of North Carolina at Chapel Hill Chapel Hill, NC United States; ^5^ Caktus Group Durham, NC United States

**Keywords:** mHealth, mobile apps, HIV, medication adherence, youth, men who have sex with men, transgender persons, games, randomized controlled trial

## Abstract

**Background:**

In the United States, young men who have sex with men (YMSM) and transgender women who have sex with men (YTWSM) bear a disproportionate burden of prevalent and incident HIV infections. Once diagnosed, many YMSM and YTWSM struggle to engage in HIV care, adhere to antiretroviral therapy (ART), and achieve viral suppression. Computer-based interventions, including those focused on behavior change, are recognized as effective tools for engaging youth.

**Objective:**

The purpose of the study described in this protocol is to evaluate the efficacy of Epic Allies, a theory-based mobile phone app that utilizes game mechanics and social networking features to improve engagement in HIV care, ART uptake, ART adherence, and viral suppression among HIV-positive YMSM and YTWSM. The study also qualitatively assesses intervention acceptability, perceived impact, and sustainability.

**Methods:**

This is a two-group, active-control randomized controlled trial of the Epic Allies app. YMSM and YTWSM aged 16 to 24 inclusive, with detectable HIV viral load are randomized 1:1 within strata of new to care (newly entered HIV medical care ≤12 months of baseline visit) or ART-nonadherent (first entered HIV medical care >12 months before baseline visit) to intervention or control conditions. The intervention condition addresses ART adherence barriers through medication reminders and adherence monitoring, tracking of select adherence-related behaviors (eg, alcohol and marijuana use), an interactive dashboard that displays the participant’s adherence-related behaviors and provides tailored feedback, encouragement messages from other users, daily HIV/ART educational articles, and gamification features (eg, mini-games, points, badges) to increase motivation for behavior change and app engagement. The control condition features weekly phone-based notifications to encourage participants to view educational information in the control app. Follow-up assessments are administered at 13, 26, and 39 weeks for each arm. The primary outcome measure is viral suppression. Secondary outcome measures include engagement in care, ART uptake, ART adherence, and psychosocial barriers to engagement in care and ART adherence, including psychological distress, stigma, and social support.

**Results:**

Baseline enrollment began in September 2015 and was completed in September 2016 (n=146), and assessment of intervention outcomes continued through August 2017. Results for primary and secondary outcome measures are expected to be reported in ClinicalTrials.gov by April 30, 2018.

**Conclusions:**

If successful, Epic Allies will represent a novel adherence intervention for a group disproportionately impacted by HIV in the United States. Adherent patients would require less frequent clinic visits and experience fewer HIV-related secondary infections, thereby reducing health care costs and HIV transmission. Epic Allies could easily be expanded and adopted for use among larger populations of YMSM and YTWSM, other HIV-positive populations, and for those diagnosed with other chronic diseases such as diabetes and hypertension.

**Trial Registration:**

ClinicalTrials.gov NCT02782130; https://clinicaltrials.gov/ct2/show/NCT02782130 (Archived by Webcite at http://www.webcitation.org/6yGODyerk)

## Introduction

### Background

Men who have sex with men (MSM) account for nearly two-thirds of all new HIV infections in the United States and young MSM (YMSM) are the only risk group experiencing an increase in HIV incidence [1-3]. Regional studies suggest that HIV prevalence among transgender women is among the highest of all risk groups, especially among transgender women of color, and African American transgender women in particular [4-6]. Although likely underestimated, HIV prevalence among young transgender women, including young transgender women who have sex with men (YTWSM), ranges from 4.5% to 15.9% [7]. Youth diagnosed with HIV must adjust to living with a highly stigmatized health condition that requires lifelong medical management. Due to structural, developmental, and psychosocial barriers, many youth struggle to enter medical care, initiate antiretroviral therapy (ART), adhere to ART, or achieve viral suppression (VS) [8,9]. For YMSM and YTWSM who may already be ostracized from families and friends because of their sexual identity, receiving an HIV diagnosis can lead to an increase in social isolation, as well as negative affective states such as depression and anxiety, which may create additional barriers to HIV treatment [10-13]. Interventions for YMSM and YTWSM that increase engagement in care, ART uptake, ART adherence, and VS are needed to maximize the individual and public health benefits of treatment [14].

Computer-based interventions (CBIs), particularly those delivered online, can address some of the barriers that HIV-positive youth face in engaging in traditional face-to-face interventions, such as stigma, lack of social support, time, and transportation [15,16]. A growing body of scientific literature demonstrates equivalent outcomes from in-person and CBIs across a range of health behaviors [17-27]. Youth in particular are highly receptive to CBIs and as a result, CBIs have been widely advocated in the fields of adolescent health education and prevention [21,22,28-33].

As of January 2017, 88% of US adults are online, 95% have a cell phone, and 77% have a smartphone [34,35]. Youth (ages 18-29 years) have the highest levels of smartphone ownership at 92% [35]. US lesbian, gay, bisexual, and transgender individuals under the age of 35 years have had consistently higher rates of smartphone ownership than their general population counterparts [36,37]. In addition to increased smartphone ownership, the use of mobile phone apps is on the rise [38].

Serious games (games designed to accomplish a purpose, such as influencing learning, civic engagement, or health behavior change) are increasingly being used to address behavioral and psychological factors that inhibit adherence to medical treatment regimens [39-41]. Such games are intended to be goal-oriented, immersive, challenging, and motivating [42]. Games designed to improve health can influence health attitudes and improve behavior change self-efficacy [43-46]. As a result, games are an ideal platform to engage youth in behavior change as they have the ability to attract and maintain attention, avoiding the development of boredom and attrition [42]. The ability to add “fun” into design and game play serves to enhance overall motivation.

Social networking sites are also extremely popular among young adults. As of July 2015, 90% of black and 95% of white youth aged between 18 and 29 years use social networking sites [47]. MSM and transgender women have high rates of social networking use [6], in part, because online venues often represent one of a limited number of venues for connecting with one another. Social networking has been used successfully to change behaviors, increase social support, and reduce social isolation in HIV prevention and care interventions [48,49].

Epic Allies was developed based on the information, motivation, and behavioral (IMB) skills model [50] to address the urgent need for interventions that improve engagement in care, ART uptake, and ART adherence among YMSM and YTWSM. The app was created using an iterative process with input from the target population at each stage of development to ensure acceptability, relevance, and appeal [16]. We anticipate that the gaming features will enhance motivation for behavior changes related to engagement in care and ART adherence. Furthermore, social networking features will increase motivation by providing users with a sense of community and social support. Funded by the National Institutes of Health, we worked with programmers and designers at Caktus Consulting Group, LLC to develop and test the Epic Allies prototype and found it to be acceptable among a sample of HIV-positive YMSM [16].

### Aims and Objectives

The aim of this paper is to describe the study protocol for the randomized controlled trial (RCT) of the Epic Allies intervention. The first objective of the study is to test the efficacy of the Epic Allies intervention among HIV-positive YMSM and YTWSM by conducting a two-arm RCT. The primary outcome measure is VS. Secondary outcomes include engagement in HIV care (ie, completion of HIV-related care clinic visit in last 3 months), ART uptake (ie, initiation of ART in the last 3 months), ART adherence (ie, >90% of doses taken in previous week), and psychosocial barriers to engagement in care and ART adherence such as psychological distress, stigma, and social support.

The second objective is to qualitatively assess intervention acceptability, perceived impact, and potential for long-term sustainability. In-depth interviews with a subset of intervention arm participants conducted after the intervention period will evaluate acceptability of Epic Allies and examine participants’ perspectives on the relationship between app use and study outcomes and potential for long-term sustainability of app use.

## Methods

### Trial Design

**Figure 1:**
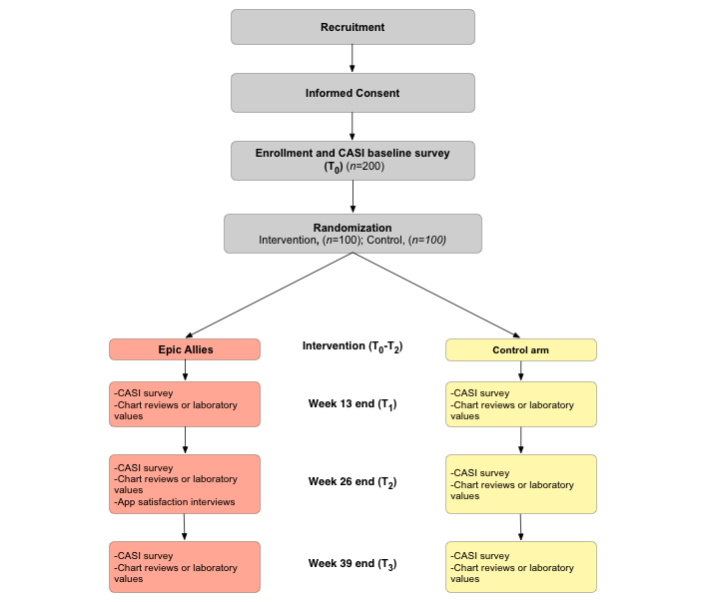
Epic Allies study schema. CASI: computer-assisted self-interviewing.

This study is a two-arm parallel RCT that will test the 26-week Epic Allies intervention against a control condition that includes weekly phone-based notifications to encourage participants to view educational information in the control app (Figure 1). Approximately 200 YMSM and YTWSM will be enrolled from 5 participating sites that provide HIV medical care for youth. Participants will be randomized 1:1 to intervention or control arms that are balanced by new to care (newly entered HIV medical care within 12 months of baseline visit) or ART-nonadherent status (first entered HIV medical care more than 12 months before baseline visit). Outcomes of interest will be measured at baseline, week 13 (during intervention phase), week 26 (end of intervention phase), and week 39 (postintervention phase). In-depth qualitative app satisfaction interviews will be conducted with approximately 20 intervention arm participants at the end of intervention use at week 26 to assess intervention experiences, acceptability, perceptions of associations between app use and study outcomes, and potential for long-term sustainability of using the app to support ART adherence.

### Ethics

The study protocol was approved by the institutional review boards (IRB) at the University of North Carolina at Chapel Hill and all participating study sites, including University of South Florida, Tampa, FL; Stroger Hospital of Cook County, Chicago, IL; Montefiore Medical Center, Bronx, NY; Tulane Medical Center, New Orleans, LA; and University of North Carolina Hospital, Chapel Hill, NC (also includes Regional AIDS Interfaith Network, Charlotte, NC). Individuals who express interest in the study will be required to provide signed informed consent before medical records are abstracted to confirm eligibility or study procedures are performed. The informed consent documents will describe all study procedures in detail. During the informed consent process, site study staff will go over the consent documents and answer any questions that may arise. A waiver of parental consent for individuals younger than 18 years has been obtained for all sites given that this is a minimal-risk study.

### Participants

Individuals participating in this study must meet the following eligibility criteria: (1) HIV-1 infected; (2) aged from 16 to 24 years; (3) assigned male sex at birth, of any gender identity, and self-reports a desire to engage or is engaging in sex with men; (4) at least one VL collected within the 12 weeks before the baseline visit, and the VL collected closest to the baseline visit is greater than the lower limit of detection for the site-specific assay used to test the specimen; (5) reliable daily access to an Android- or iOS-based mobile phone with a data plan; and (6) able to speak and read English. Self-reported eligibility criteria will be verified through an in-person screening with site study staff. Medical eligibility criteria will be verified through medical chart abstraction by site study staff. Individuals who cannot provide consent due to active substance use or psychological condition will be considered ineligible.

### Recruitment and Enrollment

Potential study participants will be identified through medical chart reviews and/or medical provider referrals at participating sites. Identified individuals will be informed of the nature of the study, the information to be collected, and the evaluations and assessments that are involved. For individuals interested in study participation, self-reported eligibility criteria will be verified. Before confirmation of medical eligibility criteria, a signed informed consent will be obtained. Individuals who provide informed consent and meet all study eligibility criteria will be enrolled in the study and complete a baseline computer-assisted self-interviewing (CASI) survey.

### Randomization

Study participants will be classified as either new to care (newly entered HIV medical care within the 12 months before the baseline visit) or ART nonadherent (first entered HIV medical care more than 12 months before the baseline visit). Randomization will occur in a 1:1 ratio within each of the 2 classification strata separately, with an equal number of participants assigned to the intervention and control arms. Due to rolling enrollment, block randomization will be used to help ensure balance within strata. Status as new to care vs nonadherent (eg, eligibility group) will be chosen as the primary stratum and randomized separately in blocks of 4 participants. Study statisticians will not be blinded to study arm assignment because they will be involved in data quality control and quality assurance.

### Incentives

The amount of participant compensation for study participation is determined separately by each site and approved by each site’s IRB. Participants will receive US $40 to $60 for completion of each RCT assessment at baseline, week 13, week 26, and week 39. Intervention arm participants who log on to the Epic Allies app 12 out of the first 14 days after the baseline visit will receive US $20 as a one-time sign-on bonus and those who log on at least once in each 30-day period will receive US $10 to help defray costs associated with smartphone data usage during that period. Participants in the intervention arm who are selected for and complete the in-depth qualitative interview will receive US $20 to $50.

### Intervention Theoretical Model and Features

**Figure 2:**
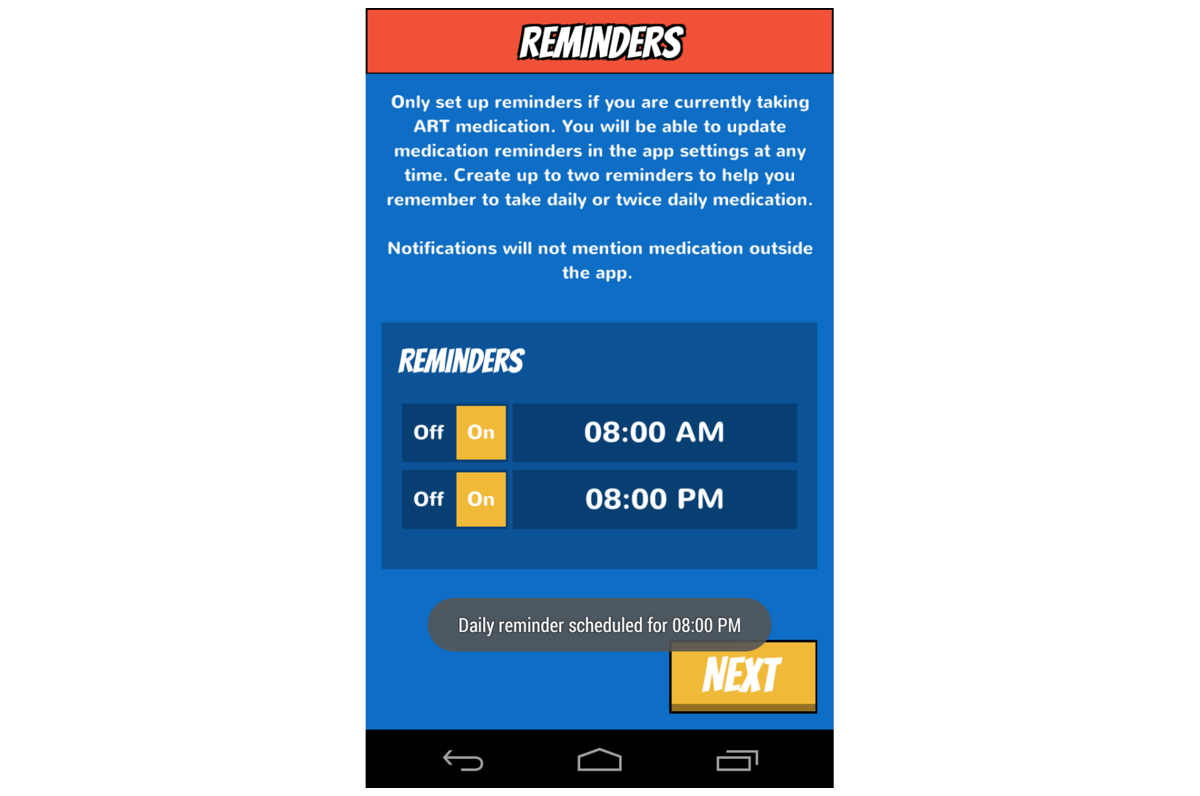
Medication reminder setup. ART: antiretroviral therapy.

**Figure 3:**
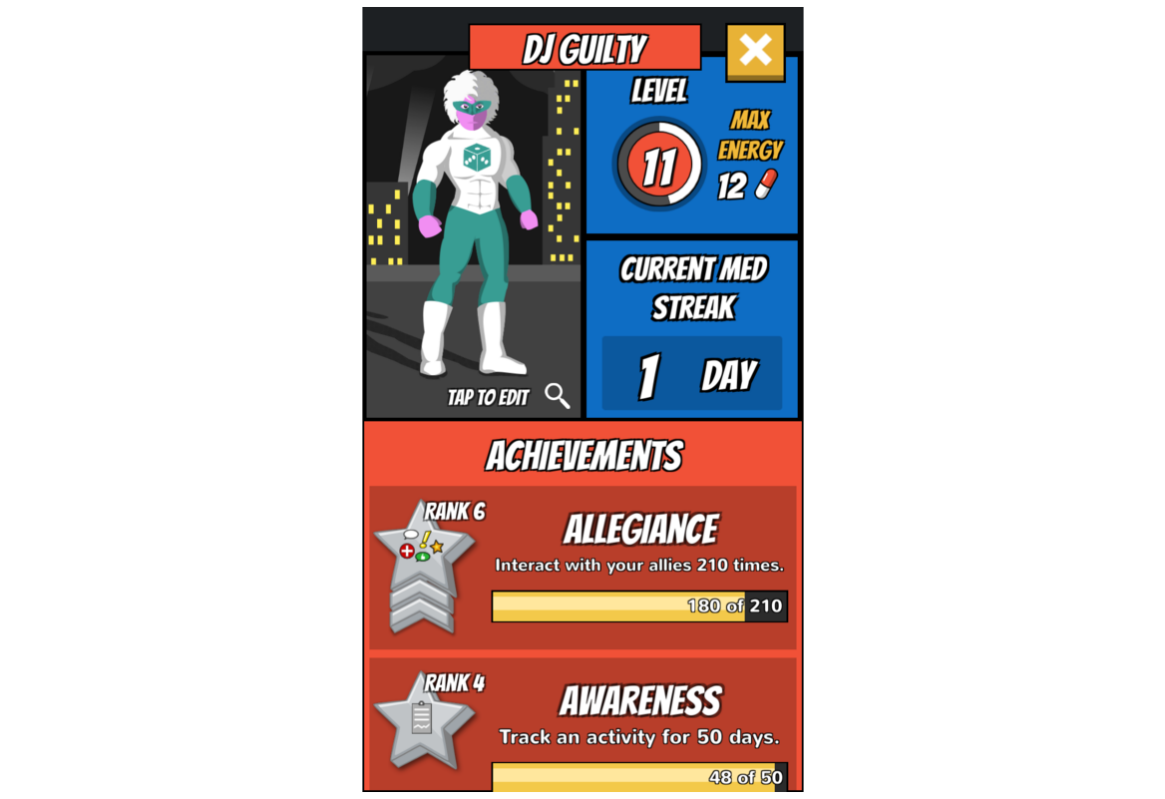
Profile.

**Figure 4:**
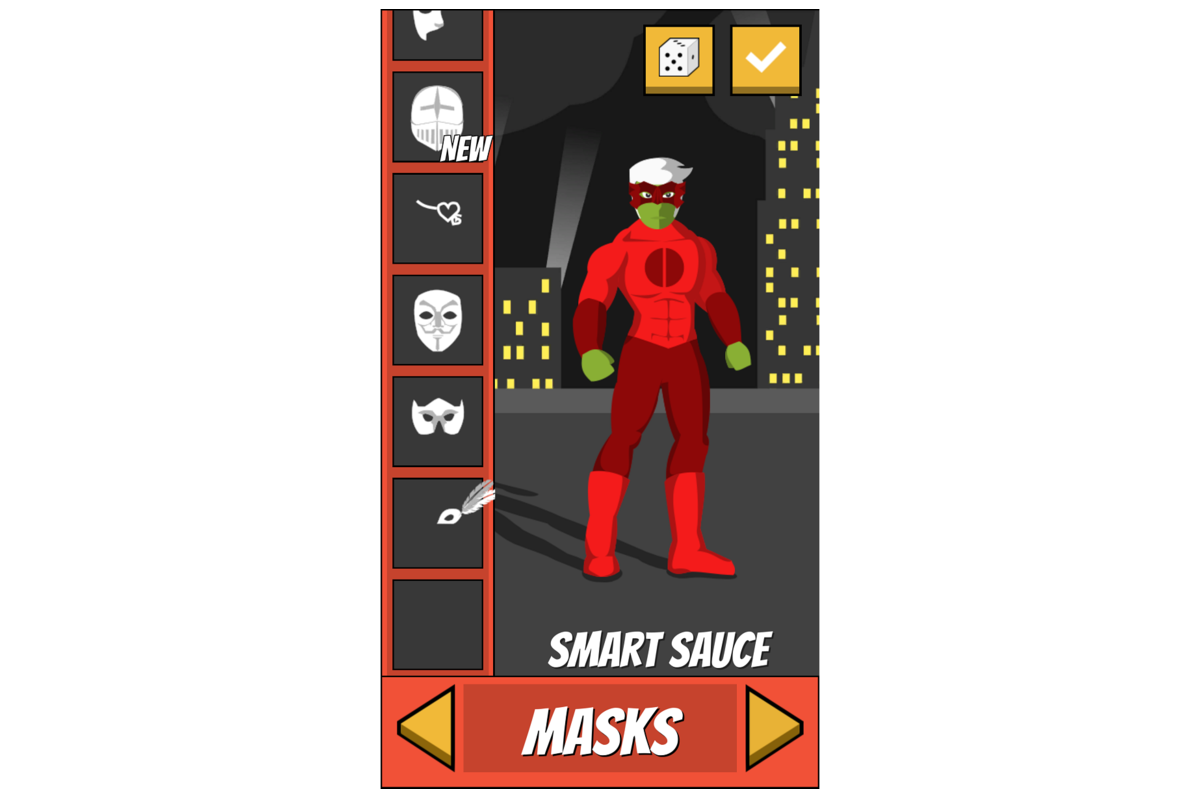
Profile: Customizable avatar.

**Figure 5:**
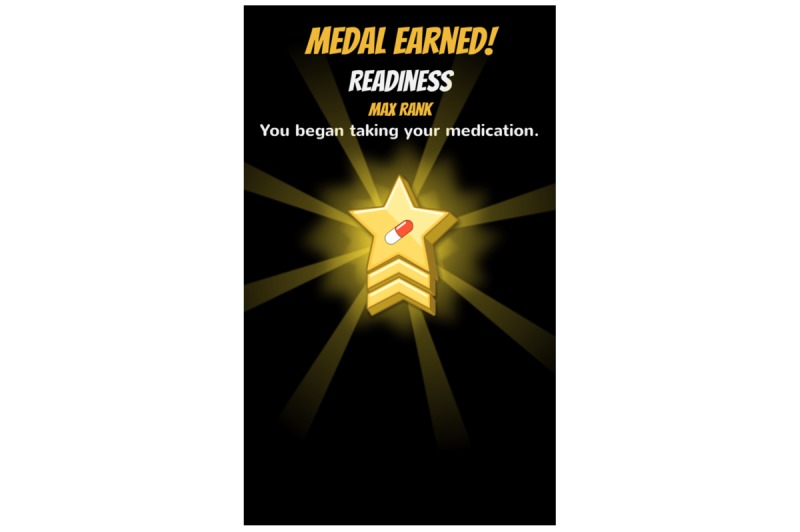
Profile: Readiness badge.

**Figure 6:**
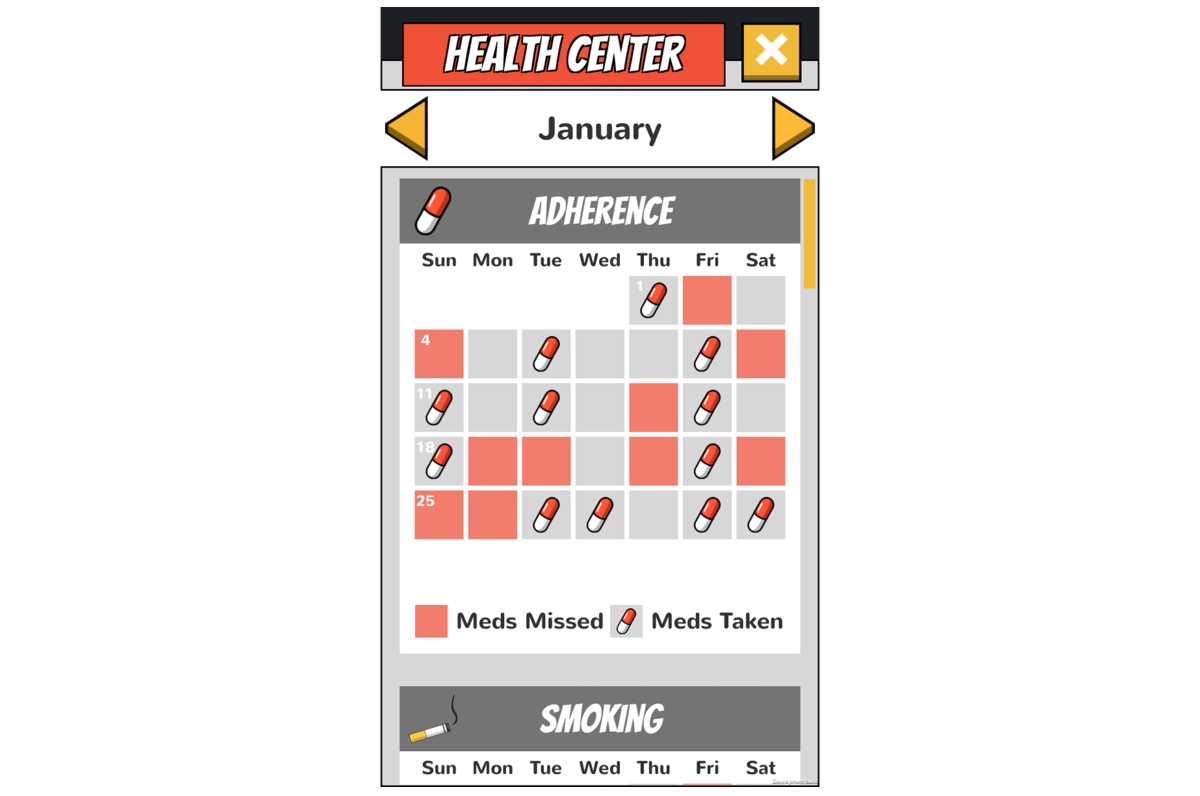
Health Center: Visual representation of adherence.

**Figure 7:**
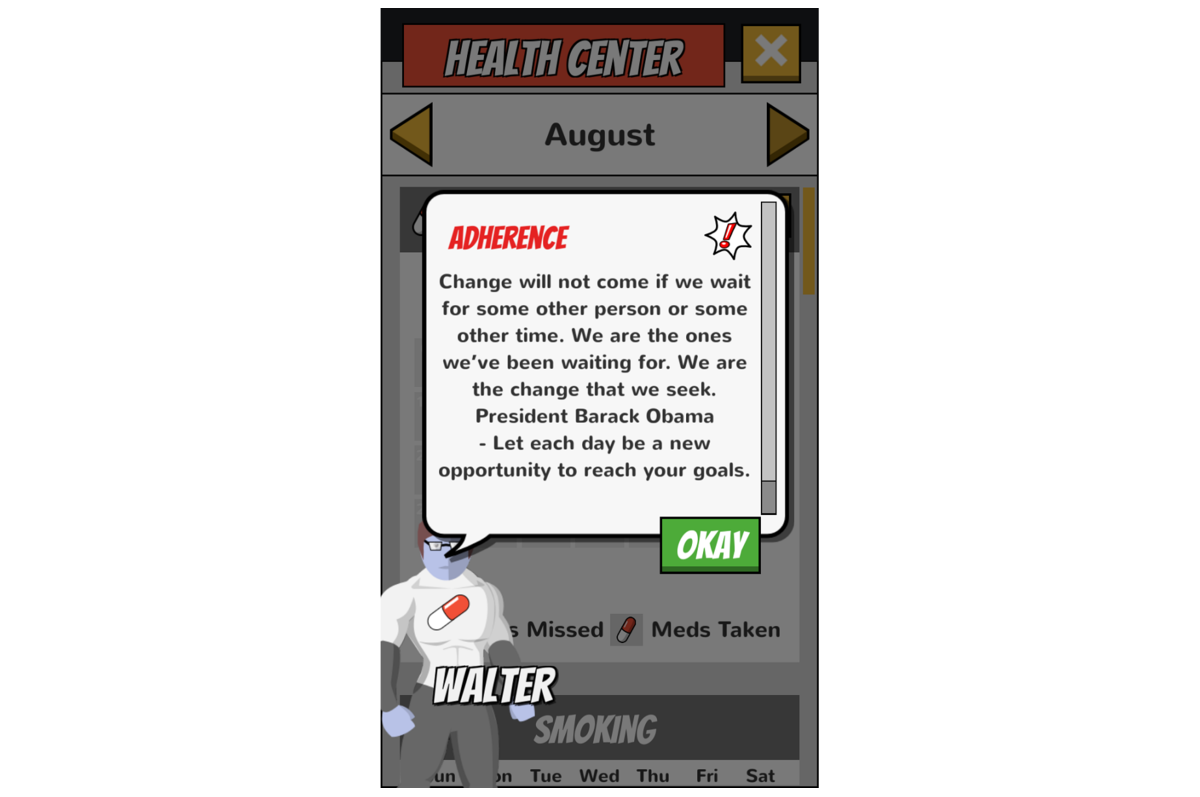
Health Center: Weekly tailored feedback.

**Figure 8:**
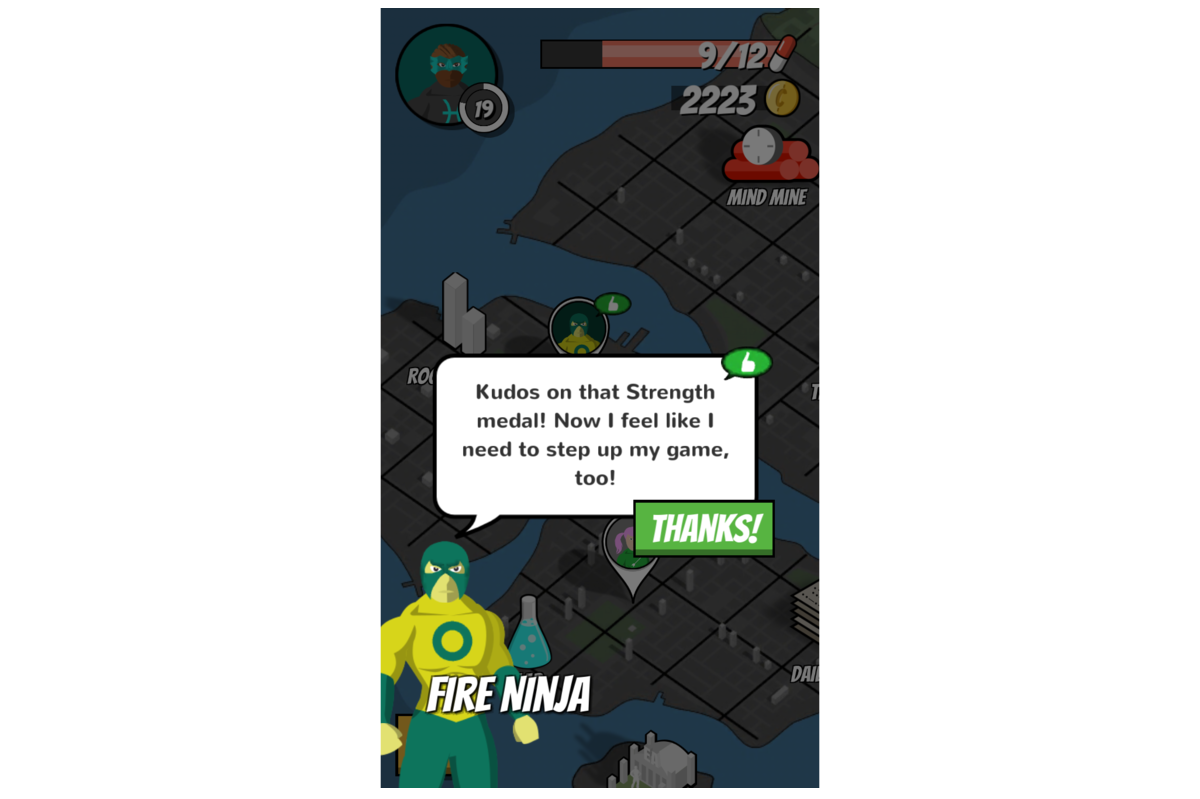
Ally interactions.

**Figure 9:**
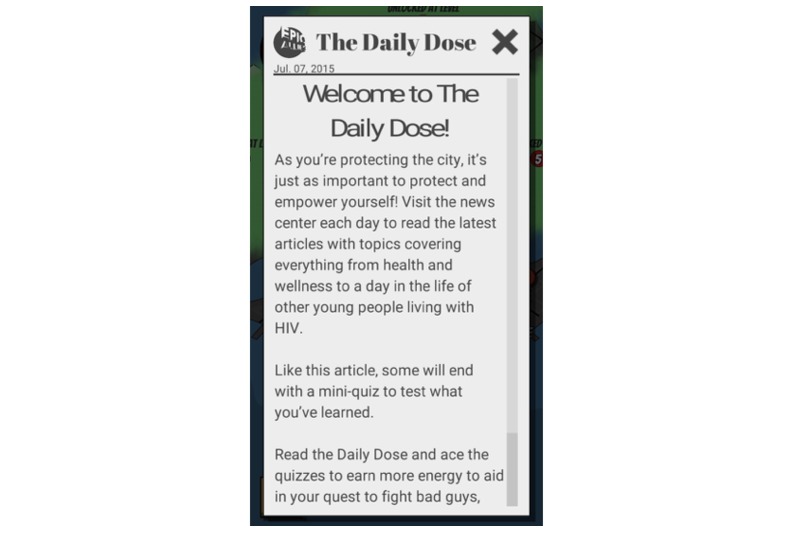
The Daily Dose.

**Figure 10:**
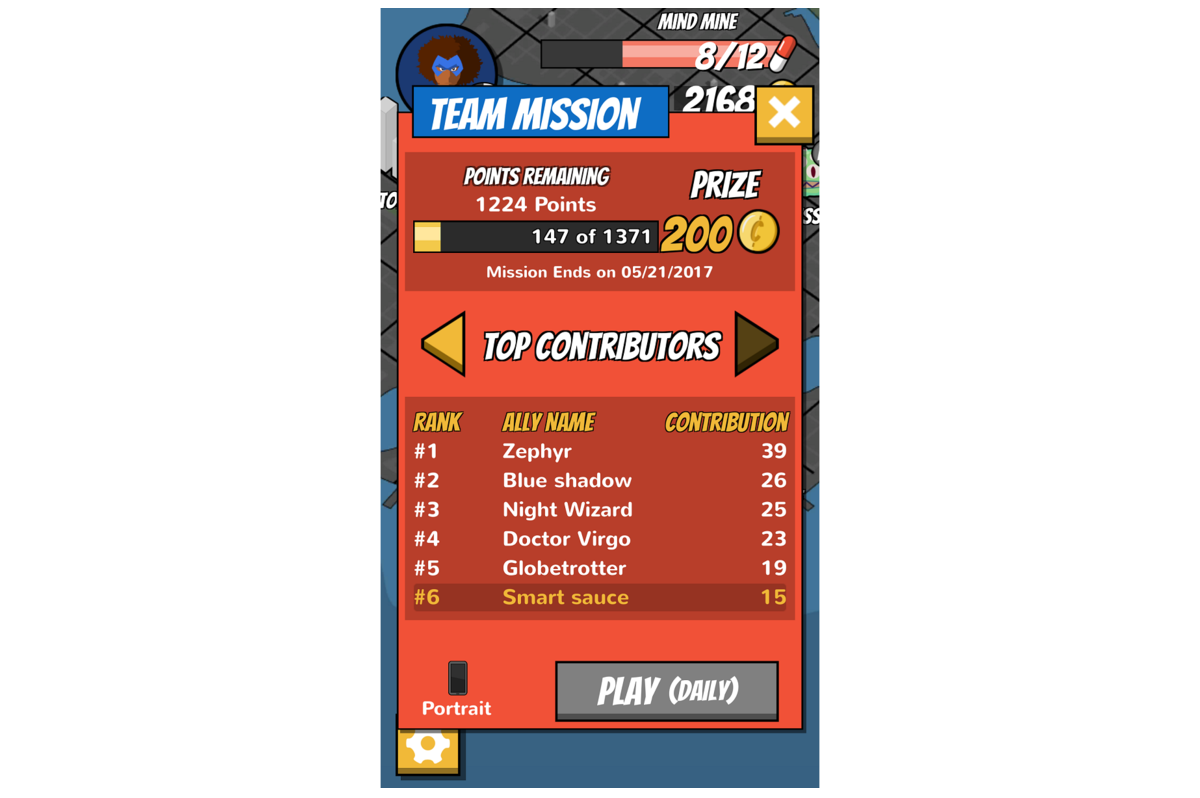
Mini-games: Social game leaderboard.

**Figure 11:**
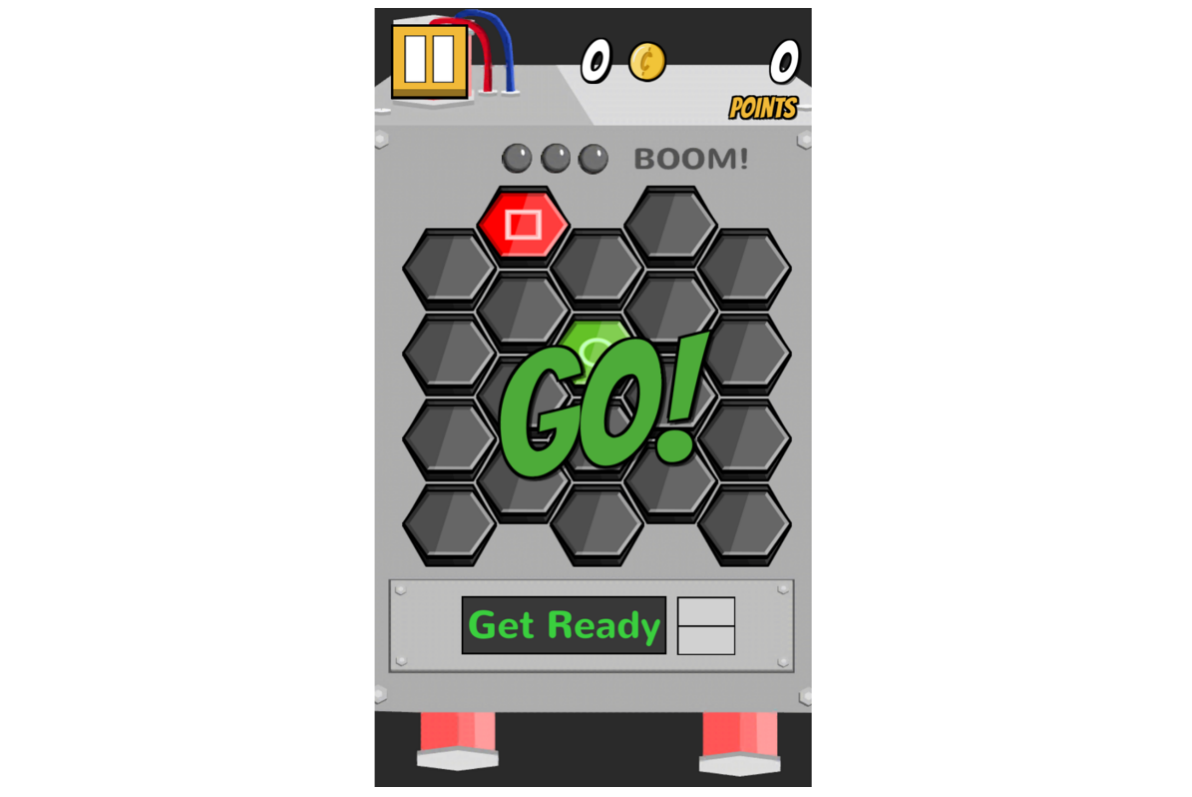
Mini-games: Mind Mine.

The design of Epic Allies was informed by the IMB skills model, which conceptualizes health behavior change (eg, medication adherence) as a product of mediators, including information about the behavior, motivation to change, and the skills needed to achieve change [50]. Studies testing the IMB model of ART adherence support relationships between information, motivation, and behavioral skills and medication adherence [51-55]. Group- and individual-level IMB-based interventions improve ART adherence [56-59], though none have been designed specifically for YMSM and YTWSM. Epic Allies’ features (see Multimedia Appendix 1) address numerous elements of the IMB model (Figures 2-11). For example, the gaming features are designed to enhance sustained app use and motivate patterning new adherence behaviors [60]. The social networking features allow users to give and receive support, a relevant need for many YMSM and YTWSM who experience social isolation due to HIV-related stigma and homophobia [61-63].

### Intervention and Control Conditions

At baseline visit, participants assigned to the intervention arm will download and install Epic Allies, create a 4-digit app password, and receive a guided tour of the app by site study staff. Intervention arm participants will have full access to all features of Epic Allies during the 26-week intervention period. Participants assigned to the control group will download and install the Epic Allies control app (phone notification messages only), create a 4-digit app password, and be provided with instructions on using the app. During the 26-week trial, control participants will receive weekly phone notifications that inform users that new content is available and one brief informational article will be provided. Control group articles are a subset of Daily Dose articles focused on ART adherence and HIV disease self-management.

### Data Collection Study Objective 1: Efficacy

Baseline, week 13, week 26, and week 39 assessments will be conducted in person. At each time point, participants will complete a CASI survey. Clinical data will include data collected via chart abstraction and/or laboratory values (VL only). If a participant does not have a VL value recorded in their chart in the 6-week window before the study visit, VL testing will be conducted on the day of the study visit as part of standard of care (ie, participant is scheduled for a medical care visit that includes VL testing) or by the study (ie, the study visit does not coincide with a medical care visit with VL testing). Table 1 lists primary and secondary outcomes and the source, collection points, and a description of each measure.

App usage data will be transmitted from the participant’s smartphone to a secure server any time the participant is connected to the Internet via broadband or Wi-Fi. App data metrics include log-ins/log-outs, use of app features, and app progress.

### Data Collection Study Objective 2: Acceptability

In-depth qualitative app satisfaction interviews will be conducted via Skype with approximately 20 intervention arm participants at the end of the intervention. We will attempt to enroll equal numbers of participants into one of four cells in Table 2 based on their ART status at study entry and app usage during the intervention. As each participant finishes the 26-week intervention period, they will be asked if they are willing to participate in the in-depth app qualitative satisfaction interview. Once a given care-usage cell has reached its quota, that cell will be “closed,” and interviews will be offered to only those participants who fall within the remaining open cells.

The in-depth qualitative app satisfaction interview will last between 45 and 60 min and will be recorded with the participant’s consent. Participants who prefer can opt to use video during the interview, but there will be no video recording. All interviews will be conducted by one of 3 trained qualitative interviewers from the study team using a semistructured interview guide. Following each interview, the recording will be transcribed by Verbal Ink (a division of Ubiqus, Los Angeles, California), checked for accuracy by study staff, and uploaded to the UNC-CH secure server. The transcripts of the first three interviews will be reviewed by the study team to assess for quality and content before completing the remaining interviews.

**Table 1:** Primary and secondary outcomes. “X” indicates that this outcome was assessed at the time point indicated in the column above. VS: viral suppression. VL: viral load. CASI: computer-assisted self-interviewing. ART: antiretroviral therapy.

Outcome		Source	13 weeks	26 weeks	39 weeks	Description
**Primary study outcomes**						
	VS defined as VL below the lower limit of detection in the 6-week window before the scheduled study visits	Chart review (any value in 6-week window before scheduled visit) OR laboratory value collected at study visit	X	X		<40 copies/mL or lower limit of detection for site-specific assay used to test the specimen
**Secondary study outcomes**						
	VL suppression defined as VL below the lower limit of detection in the 6-week window before the scheduled study visits	Chart review (any value in 6-week window before scheduled visit) OR laboratory value collected at study visit			X	<40 copies/mL or lower limit of detection for site-specific assay used to test the specimen
	Engagement in care	CASI survey	X	X	X	Completion of HIV-related care clinic visit in last 3 months
	ART uptake (for participants not on ART)	CASI survey	X	X	X	“Are you currently taking medication to treat your HIV (Y/N)?”
	ART adherence^a^ (for participants on ART)	CASI survey	X	X	X	(1) “How many times during the day has your doctor told you to take a dose of medicine (pills or other medicines) to treat your HIV?” and (2) “Thinking about the last 7 days, how many times did you miss taking a dose of pills?”[64]

^a^Outcome of >90% adherence is comprised 2 components (1) is the denominator, indicating the frequency of doses prescribed (multiplied x7 to represent total weekly doses); (2) is the numerator, indicating the number of times, total, a dose was missed.

**Table 2:** In-depth qualitative app satisfaction interview enrollment.

Antiretroviral therapy (ART) experience at entry		New to care	ART nonadherent	Total
**App utilization pattern**				
	Intervention low users (uses app <4 days/week)	5	5	10
	Intervention users (uses app ≥4 days/week)	5	5	10
Total		10	10	20

### Follow-Up and Retention

#### Tracking Participant Follow-Up

All participants will be contacted before each follow-up study visit (ie, 13, 26, and 39 weeks after baseline). Multiple contact methods will be used for youth who are difficult to reach (eg, mail, alternate phone numbers, email, text message, Facebook). Participants will be asked whether or not messages can be left for each of the phone numbers that they provide. They will be informed that messages will not contain any information regarding the nature of the project.

#### Study Visit Management

The preferred time frame for all follow-up visits is within 4 weeks before or after the target study visit date. If the participant is unable to attend a visit within this time frame, the site staff will work with the participant to identify a day closest to the scheduled visit to perform the visit.

Participants in the intervention arm will be reminded by the Epic Allies app via a discreet phone notification (eg, “Your allies need you–log in to Epic Allies”) to log on to the app every week. If a participant does not log on for 4 weeks, study staff will notify site staff and ask that they reach out to the participant.

#### Completing Web-Based Computer-Assisted Self-Interviewing Surveys

CASI surveys will ideally be completed at the clinic site during each study visit. Participants will be provided with a quiet, private area to complete the survey. The survey may be completed on the participant’s smartphone, but a computer with Wi-Fi connection should be made available in case the participant prefers to complete the survey on a computer.

If a participant is unable to attend a follow-up study visit, the participant may complete the survey on his or her own. The survey should be completed within 4 weeks before or after the study visit target date. If a participant is unable to complete the survey within this window due to extenuating circumstances, the window may be lengthened to 7 weeks.

#### Data Security

##### Epic Allies App Data

Caktus Consulting Group will store app usage data on a secure Web server for the duration of the study. At the end of the study, Caktus Consulting Group will send app usage data to the study team, destroy the data on the server, and then shut down the server. Protected health information is neither collected nor stored on the Web server.

##### In-Depth Qualitative App Satisfaction Interview

For the in-depth qualitative app satisfaction interview with intervention arm participants, the audio recording as well as the transcript will be marked with the participant’s study ID only. Any identifying information mentioned in the interviews will be redacted in the transcripts, thus the transcripts will be deidentified. Both files will be uploaded and stored on a secure server.

### Data Analysis Study Objective 1: Efficacy

#### Sample Size and Power Estimates

We estimated a sample size of 200 as feasible enrollment. Power calculations are estimated to detect between-group differences in the primary outcome (viral suppression) in a parallel two-group repeated measures design with equal allocation, based on a generalized estimating equation (GEE) framework assuming an exchangeable covariance structure, measurements at 3 follow-up points, and correlation among same participant repeat measures (rho) of .4. For all estimates, we used a two-sided test of significance and an alpha level of .05. Assuming a 20% loss to follow-up, we will have 80% power to detect absolute differences in viral suppression of 16.3% between the intervention and control groups in the proportion of participants with viral suppression when the proportion in the control group is 27%.

#### Quantitative Data Analysis

We will compare within- and between-group differences in primary and secondary outcomes for each follow-up time period. The 13- and 26-week follow-up will be considered our primary endpoint for the primary outcome, thus *P* values will only be computed for these time periods for the primary outcome. *P* values will be adjusted for multiple comparisons using the Benjamini-Hochberg procedure [65]. Estimates for all outcomes will be presented with 95% CI. Intervention and control groups will be compared on baseline characteristics to assess balance. Patterns of missing data for our primary outcome of VS will be examined and baseline characteristics of participants with complete vs incomplete follow-up will be compared with assess nonresponse and attrition biases.

Intervention effects will be evaluated using an intention-to-treat (ITT) approach. The primary study outcome (VS, defined as the lower limit of detection of site assay) will be compared at 13, 26, and 39 weeks in the intervention and control groups using generalized linear models (GLM), which can be used for dependent variables with normal, binary, poisson, and negative binomial distributions. Link functions will be selected as appropriate based on the distribution of the dependent variable. We will apply the GEE extension of GLM to account for within-participant correlation associated with repeated measures. GEEs allow for inclusion of categorical and count-dependent variables and appropriate specification of working covariance structures for observations that are correlated within groups and across time. Fixed main effects parameters for study site and eligibility group will be fitted to data to account for the nature of the randomized design. Intervention efficacy will be assessed in terms of the main effect for overall group differences. Use of a GEE framework means that inference will be made to the marginal effect of the Epic Allies treatment on the outcome, averaged across the study population. Secondary analyses will be performed similar to the methods described above to identify potential mediators and moderators of the intervention impact on primary outcome.

#### Missing, Unused, and Spurious Data

Several procedures will be used to conduct data analysis when data for either outcomes or baseline covariates are missing. The first step will be to assess the extent and pattern of missing data. If data are missing for only a few cases, then data analysis will be conducted only on study participants with complete data. If the pattern of missing outcome data is monotone, then inverse probability weighting will be performed to adjust the available data for loss to follow-up [66]. If substantial nonmonotone missing outcome data are present, then a multiple imputation approach will be used. Unused or spurious data will be documented and discussed when disseminating results of this study. Baseline covariates will be compared between participants with complete follow-up vs those who have incomplete follow-up in order to assess the presence of informative missingness.

### Data Analysis Study Objective 2: Acceptability, Perceived Impact, and Sustainability

#### Qualitative Approach

The interview and analysis structure will follow a phenomenological approach to optimize our ability to capture and understand the study’s experience-based topics of interest (eg, experience of HIV diagnosis and acceptance, experience of engaging with Epic Allies intervention and participants). Phenomenology is an ideal theoretical approach for this component as it is focused on describing both *what* a given group of participants experience and *how* they experience this particular phenomenon [67-69]. Data are presented through textual descriptions of the phenomena based on summaries of the experiences described by respondents. The composite descriptions offer an explanation of the underlying structure which exists across the participants’ experiences [69,70]. This will focus on individual and shared experiences and meanings.

#### Qualitative Data Analysis

For the analysis, process interviews will be transcribed and then we will begin with our a priori list of themes (experience using Epic Allies, recent ART adherence challenges, etc). Study team members will read all transcripts and identify emergent themes from participants’ experiences. These themes will be discussed as a group, and a final list of themes will be developed with brief descriptions, relationships between themes, and supporting quotes. For the qualitative research component, the Atlas.ti qualitative data analysis software (version 8, Scientific Software Development, Berlin, Germany) will be used to assist with theme identification and building, as well as coding textual data [69,71]. Coding and analytic activities will be discussed during weekly team meetings.

### Interim Analysis

No interim analysis will be performed for this study. The study team determined that this study does not involve greater than minimal risk (45 CFR Part 46.404 and 21 CFR Part 50.51). Participation in this study poses no more harm or discomfort to participants than they may experience in normal daily life or during routine physical or psychological examinations or tests.

### Protection Against Harms

All sites have specific policies governing the treatment of human subjects. These policies specify that medical and psychological assistance will be available in the immediate environment in the event a participant should experience any adverse reactions resulting from study procedures.

## Results

A total of 146 YMSM and YTWSM were enrolled in Epic Allies between September 2015 and 2016. Demographic characteristics of study participants are shown in Table 3. Although we estimated 200 as feasible for enrollment, study sites had fewer individuals eligible for participation in the study than expected. As a result, our ability to detect differences in our primary outcome (viral suppression) with 80% power assuming 20% loss to follow-up and the proportion of viral suppression in the control group is 27%, decreases by 3.1% (>16.3% to >19.4%).

**Table 3:** Sample characteristics of Epic Allies study population by intervention arm. Q1: 25^th^ percentile. Q3: 75^th^ percentile.

Characteristic		Intervention (N=74)	Control (N=72)	Total (N=146)
**Classification strata, n (%)**				
	New to care	36 (49)	38 (53)	74 (50.7)
	Antiretroviral therapy nonadherent	38 (51)	34 (47)	72 (49.3)
**Study site, n (%)**				
	University of South Florida	19 (26)	11 (15)	30 (20.5)
	Stroger Hospital	9 (12)	14 (19)	23 (15.8)
	Montefiore Medical Center	15 (20)	15 (21)	30 (20.5)
	Tulane Medical Center	9 (12)	17 (24)	26 (17.8)
	University of North Carolina	22 (30)	15 (21)	37 (25.3)
**Age <18 years, n (%)**				
	No	71 (96)	66 (92)	137 (94.0)
**Age**				
	Median	22.0	21.0	21.5
	Q1, Q3	20.0, 23.0	20.0, 23.0	20.0, 23.0
**Gender identity, n (%)**				
	Male	69 (93)	67 (93)	136 (93.2)
	Transgender female	5 (7)	3 (4)	8 (5.5)
	Other	0 (0)	2 (3)	2 (1.4)
**Sexual identity, n (%)**				
	Gay	55 (74)	58 (81)	113 (77.4)
	Bisexual	16 (22)	11 (15)	27 (18.5)
	Other	3 (4)	3 (4)	6 (4.1)
**Hispanic or Latino ethnicity, n (%)**				
	Yes	17 (23)	12 (17)	29 (19.9)
	No	57 (77)	60 (83)	117 (80.1)
**Black or African American race, n (%)**				
	Yes	60 (81)	60 (83)	120 (82.2)
	No	14 (19)	12 (17)	26 (17.8)
**White race, n (%)**				
	Yes	10 (14)	6 (8)	16 (11.0)
	No	64 (86)	66 (92)	130 (89.0)
**Other race, n (%)**				
	Yes	5 (7)	8 (11)	13 (8.9)
	No	69 (93)	64 (89)	133 (91.1)
**Highest level of education completed, n (%)**				
	<12th grade	17 (23)	11 (15)	28 (19.2)
	Completed high school/General Equivalency Diploma, some technical school/college	48 (65)	58 (81)	106 (72.6)
	College/technical degree or more	9 (12)	3 (4)	12 (8.2)
**Annual income, n (%)**				
	<US $11,999	56 (76)	52 (72)	108 (74.0)
	US $12,000+	10 (14)	13 (18)	23 (15.8)
	Don't know/Refuse	8 (11)	7 (10)	15 (10.3)
**Employment, n (%)**				
	Yes	51 (69)	44 (61)	95 (65.1)
	No	23 (31)	28 (39)	51 (34.9)
**Health insurance, n (%)**				
	Medicaid	24 (32)	26 (36)	50 (34.2)
	Private health insurance (eg, Blue Cross Blue Shield, parent's)	7 (9)	15 (21)	22 (15.1)
	AIDS Drug Assistance Program	21 (28)	17 (24)	38 (26.0)
	Other	6 (8)	0 (0)	6 (4.1)
	I do not have health insurance	16 (22)	14 (19)	30 (20.5)
**Homelessness in past 3 months, n (%)**				
	Yes	24 (32)	18 (25)	42 (28.8)
	No	50 (68)	54 (75)	104 (71.2)
**Lifetime incarceration, n (%)**				
	Yes	21 (28)	18 (25)	39 (26.7)
	No	53 (72)	54 (75)	107 (73.3)

## Discussion

### Epic Allies Summary

Epic Allies addresses ART uptake and adherence, a critical need among a disproportionately affected patient population via familiar technologies using engaging, theory-based components. The app targets the most common ART adherence barriers among youth, addresses specific behavior outcomes, and is tailored for the target population and customizable for individual users. The social support, encouragement, and informational features listed above are designed to help youth overcome barriers to adherence across various stages of engagement in HIV care, ranging from lack of understanding and low health literacy, coping with side effects and drug toxicities, to the impact of drug and alcohol use on ART adherence.

This novel intervention app, Epic Allies, targets HIV-positive YMSM and YTWSM, aged between 16 and 24 years (inclusive), with a detectable HIV VL. Epic Allies utilizes self-management tools, social support, and gamification to increase ART information, motivation, and behavioral skills and improve ART adherence, including (1) real-time data tracking of adherence with graphic visualizations; (2) tailored reminders and motivational messages; (3) connection to a network of other HIV-positive YMSM and YTWSM; and (4) a gaming approach engineered to reinforce daily adherence tracking, promote social networking support among users, encourage learning and skill building, and maintain user engagement.

### Limitations

As with all longitudinal studies, a loss of participants to follow-up may induce a selection bias if missingness is informative and is related to both treatment arm and the study outcome. Furthermore, if compliance with the treatment assignment is less than 100% in either study arm, the ITT estimate, the study’s primary estimate, will differ from the compliance-averaged causal estimate [72,73]. In this case, the ITT estimate will still validly measure the efficacy of being randomized to the treatment arm but may not estimate the efficacy of the treatment itself. Data for secondary outcomes will be collected primarily from self-report survey, which is prone to both exposure and outcome misclassification. This misclassification could bias our study results either toward or away from the null hypothesis. Contamination may also be an issue, as participants at each of the study sites can be randomized to the intervention or control arm, and participants in the intervention arm may show Epic Allies to those in the control arm. All study participants are recruited from sites that provide HIV medical care and have procedures to monitor and address poor retention in care. Although many study participants were not regularly engaged in care at enrollment, retention in care outcomes among the study sample may be inflated when compared with a community-recruited sample. Thus, caution should be exercised regarding generalizability of retention in care outcomes. Finally, a modest sign-on bonus for regular use of the app in the first 2 weeks of the study and nominal monthly data use reimbursements are offered to those in the intervention group but not to those in the control group. The purpose of the sign-on bonus is to encourage regular use of the app early in the study to try to increase the likelihood that app use becomes a daily habit. The bonus is intentionally modest and time-limited to decrease the likelihood that money alone influences differences in study outcomes between the arms. Reimbursement for data use is intended to ensure that intervention arm participants do not intentionally avoid the app due to concerns about data usage. This is only warranted for intervention participants because the amount of data used for the control app is extremely low. While it is important to acknowledge the differences in incentives for the intervention and control groups, we believe they are unlikely to explain differences in outcomes between the arms.

### Conclusions

If successful, Epic Allies will represent a novel adherence intervention for a group disproportionately impacted by HIV in the United States. Epic Allies would be clinically attractive, as adherent patients would require less frequent clinic visits and experience fewer HIV-related secondary infections [74-76]. Reducing clinic visits and secondary infections could make the intervention financially attractive by reducing health care costs. Epic Allies could also greatly impact public health as ART adherence reduces HIV infectivity and subsequently reduces HIV transmission [74]. Epic Allies could be used during times of adherence vulnerability (eg, when initiating ART or changing medication regimens) and could easily be expanded and adopted for use among larger populations of YMSM and YTWSM, other HIV-positive populations, and for those diagnosed with other chronic diseases such as diabetes and hypertension.

## Appendix

Multimedia Appendix 1 Features of Epic Allies.

## Abbreviations

ART: antiretroviral therapy
CASI: computer-assisted self-interviewing
CBIs: computer-based interventions
GEE: generalized estimating equation
GLM: generalized linear models
RCT: randomized controlled trial
VL: viral load
VS: viral suppression
YMSM: young men who have sex with men
YTWSM: transgender women who have sex with men

## References

[1] Prejean J, Song R, Hernandez A, Ziebell R, Green T, Walker F, Lin LS, An Q, Mermin J, Lansky A, Hall HI, HIV Incidence Surveillance Group (2011). Estimated HIV incidence in the United States, 2006-2009. PLoS One.

[2] (2011). CDC.

[3] An Q, Prejean J, Hall HI (2012). Racial disparity in U.S. diagnoses of acquired immune deficiency syndrome, 2000-2009. Am J Prev Med.

[4] Herbst JH, Jacobs ED, Finlayson TJ, McKleroy VS, Neumann MS, Crepaz N, HIV/AIDS Prevention Research Synthesis Team (2008). Estimating HIV prevalence and risk behaviors of transgender persons in the United States: a systematic review. AIDS Behav.

[5] Clements-Nolle K, Marx R, Guzman R, Katz M (2001). HIV prevalence, risk behaviors, health care use, and mental health status of transgender persons: implications for public health intervention. Am J Public Health.

[6] Sevelius JM, Keatley J, Gutierrez-Mock L (2011). HIV/AIDS programming in the United States: considerations affecting transgender women and girls. Womens Health Issues.

[7] Poteat T, Scheim A, Xavier J, Reisner S, Baral S (2016). Global epidemiology of HIV infection and related syndemics affecting transgender people. J Acquir Immune Defic Syndr.

[8] Zanoni BC, Mayer KH (2014). The adolescent and young adult HIV cascade of care in the United States: exaggerated health disparities. AIDS Patient Care STDS.

[9] Ryscavage P, Anderson EJ, Sutton SH, Reddy S, Taiwo B (2011). Clinical outcomes of adolescents and young adults in adult HIV care. J Acquir Immune Defic Syndr.

[10] Hosek SG, Harper GW, Lemos D, Martinez J (2008). An ecological model of stressors experienced by youth newly diagnosed with HIV. J HIV AIDS Prev Child Youth.

[11] Hosek SG, Harper GW, Domanico R (2015). Psychological and social difficulties of adolescents living with HIV: a qualitative analysis. J Sex Educ Ther.

[12] Lam PK, Naar-King S, Wright K (2007). Social support and disclosure as predictors of mental health in HIV-positive youth. AIDS Patient Care STDS.

[13] Murphy DA, Durako SJ, Moscicki AB, Vermund SH, Ma Y, Schwarz DF, Muenz LR, Adolescent Medicine HIV/AIDS Research Network (2001). No change in health risk behaviors over time among HIV infected adolescents in care: role of psychological distress. J Adolesc Health.

[14] Gardner EM, McLees MP, Steiner JF, Del Rio C, Burman WJ (2011). The spectrum of engagement in HIV care and its relevance to test-and-treat strategies for prevention of HIV infection. Clin Infect Dis.

[15] Lall P, Lim SH, Khairuddin N, Kamarulzaman A (2015). Review: an urgent need for research on factors impacting adherence to and retention in care among HIV-positive youth and adolescents from key populations. J Int AIDS Soc.

[16] LeGrand S, Muessig KE, McNulty T, Soni K, Knudtson K, Lemann A, Nwoko N, Hightow-Weidman LB (2016). Epic Allies: Development of a Gaming App to Improve Antiretroviral Therapy Adherence Among Young HIV-Positive Men Who Have Sex With Men. JMIR Serious Games.

[17] Feil EG, Noell J, Lichtenstein E, Boles SM, McKay HG (2003). Evaluation of an Internet-based smoking cessation program: lessons learned from a pilot study. Nicotine Tob Res.

[18] Lenert L, Muñoz RF, Stoddard J, Delucchi K, Bansod A, Skoczen S, Pérez-Stable EJ (2003). Design and pilot evaluation of an internet smoking cessation program. J Am Med Inform Assoc.

[19] Napolitano MA, Fotheringham M, Tate D, Sciamanna C, Leslie E, Owen N, Bauman A, Marcus B (2003). Evaluation of an internet-based physical activity intervention: a preliminary investigation. Ann Behav Med.

[20] Newman MG, Kenardy J, Herman S, Taylor CB (1997). Comparison of palmtop-computer-assisted brief cognitive-behavioral treatment to cognitive-behavioral treatment for panic disorder. J Consult Clin Psychol.

[21] Schinke SP, Schwinn TM, Di Noia J, Cole KC (2004). Reducing the risks of alcohol use among urban youth: three-year effects of a computer-based intervention with and without parent involvement. J Stud Alcohol.

[22] Schinke SP, Di Noia J, Glassman JR (2004). Computer-mediated intervention to prevent drug abuse and violence among high-risk youth. Addict Behav.

[23] Sciamanna CN, Lewis B, Tate D, Napolitano MA, Fotheringham M, Marcus BH (2002). User attitudes toward a physical activity promotion website. Prev Med.

[24] Selmi PM, Klein MH, Greist JH, Sorrell SP, Erdman HP (1991). Computer-administered therapy for depression. MD Comput.

[25] Tate DF, Wing RR, Winett RA (2001). Using Internet technology to deliver a behavioral weight loss program. JAMA.

[26] Tate DF, Jackvony EH, Wing RR (2003). Effects of Internet behavioral counseling on weight loss in adults at risk for type 2 diabetes: a randomized trial. JAMA.

[27] Woodruff SI, Edwards CC, Conway TL, Elliott SP (2001). Pilot test of an Internet virtual world chat room for rural teen smokers. J Adolesc Health.

[28] Hightow-Weidman LB, Pike E, Fowler B, Matthews DM, Kibe J, McCoy R, Adimora AA (2012). HealthMpowerment.org: feasibility and acceptability of delivering an internet intervention to young Black men who have sex with men. AIDS Care.

[29] Lightfoot M, Comulada WS, Stover G (2007). Computerized HIV preventive intervention for adolescents: indications of efficacy. Am J Public Health.

[30] Noar SM, Black HG, Pierce LB (2009). Efficacy of computer technology-based HIV prevention interventions: a meta-analysis. AIDS.

[31] Noar SM (2011). Computer technology-based interventions in HIV prevention: state of the evidence and future directions for research. AIDS Care.

[32] Paperny DM (1997). Computerized health assessment and education for adolescent HIV and STD prevention in health care settings and schools. Health Educ Behav.

[33] Schinke SP, Orlandi MA (1990). Skills-Based, Interactive Computer Interventions to Prevent HIV Infection Among African-American and Hispanic Adolescents. Comput Human Behav.

[34] (2017). Pew Research Center.

[35] (2017). Pew Research Center.

[36] (2012). Community Marketing Inc.

[37] (2011). Community Marketing Inc.

[38] (2015). Pew Research Center.

[39] Kato PM (2010). Video games in health care: Closing the gap. Rev Gen Psychol.

[40] Ritterfeld U, Cody M, Vorderer P (2009). Serious Games: Mechanisms and Effects.

[41] Hightow-Weidman LB, Muessig KE, Bauermeister JA, LeGrand S, Fiellin LE (2017). The future of digital games for HIV prevention and care. Curr Opin HIV AIDS.

[42] McGonigal J (2011). Reality is broken: Why games make us better and how they can change the world.

[43] Baranowski T, Baranowski J, Cullen KW, Marsh T, Islam N, Zakeri I, Honess-Morreale L, deMoor C (2003). Squire's Quest! Dietary outcome evaluation of a multimedia game. Am J Prev Med.

[44] Brown SJ, Lieberman DA, Germeny BA, Fan YC, Wilson DM, Pasta DJ (1997). Educational video game for juvenile diabetes: results of a controlled trial. Med Inform (Lond).

[45] Kato PM, Cole SW, Bradlyn AS, Pollock BH (2008). A video game improves behavioral outcomes in adolescents and young adults with cancer: a randomized trial. Pediatrics.

[46] Lieberman DA, Street Jr RL, Gold WR, Manning TR (1997). Interactive video games for health promotion: Effects on knowledge, self-efficacy, social support, and health. Health promotion and interactive technology: Theoretical applications and future directions.

[47] (2015). Pew Research Center: Internet and Technology.

[48] Hightow-Weidman LB, Muessig KE, Pike EC, LeGrand S, Baltierra N, Rucker AJ, Wilson P (2015). HealthMpowerment.org: Building Community Through a Mobile-Optimized, Online Health Promotion Intervention. Health Educ Behav.

[49] Young SD, Cumberland WG, Lee SJ, Jaganath D, Szekeres G, Coates T (2013). Social networking technologies as an emerging tool for HIV prevention: a cluster randomized trial. Ann Intern Med.

[50] Fisher JD, Fisher WA, Amico KR, Harman JJ (2006). An information-motivation-behavioral skills model of adherence to antiretroviral therapy. Health Psychol.

[51] Amico KR, Toro-Alfonso J, Fisher JD (2005). An empirical test of the information, motivation and behavioral skills model of antiretroviral therapy adherence. AIDS Care.

[52] Amico KR, Barta W, Konkle-Parker DJ, Fisher JD, Cornman DH, Shuper PA, Fisher WA (2009). The information-motivation-behavioral skills model of ART adherence in a Deep South HIV+ clinic sample. AIDS Behav.

[53] Kalichman SC, Rompa D, DiFonzo K, Simpson D, Austin J, Luke W, Kyomugisha F, Buckles J (2001). HIV treatment adherence in women living with HIV/AIDS: research based on the Information-Motivation-Behavioral Skills model of health behavior. J Assoc Nurses AIDS Care.

[54] Rongkavilit C, Naar-King S, Kaljee LM, Panthong A, Koken JA, Bunupuradah T, Parsons JT (2010). Applying the information-motivation-behavioral skills model in medication adherence among Thai youth living with HIV: a qualitative study. AIDS Patient Care STDS.

[55] Starace F, Massa A, Amico KR, Fisher JD (2006). Adherence to antiretroviral therapy: an empirical test of the information-motivation-behavioral skills model. Health Psychol.

[56] Kalichman SC, Cherry J, Cain D (2005). Nurse-delivered antiretroviral treatment adherence intervention for people with low literacy skills and living with HIV/AIDS. J Assoc Nurses AIDS Care.

[57] Margolin A, Avants SK, Warburton LA, Hawkins KA, Shi J (2003). A randomized clinical trial of a manual-guided risk reduction intervention for HIV-positive injection drug users. Health Psychol.

[58] Parsons JT, Golub SA, Rosof E, Holder C (2007). Motivational interviewing and cognitive-behavioral intervention to improve HIV medication adherence among hazardous drinkers: a randomized controlled trial. J Acquir Immune Defic Syndr.

[59] Wagner GJ, Kanouse DE, Golinelli D, Miller LG, Daar ES, Witt MD, Diamond C, Tilles JG, Kemper CA, Larsen R, Goicoechea M, Haubrich RH (2006). Cognitive-behavioral intervention to enhance adherence to antiretroviral therapy: a randomized controlled trial (CCTG 578). AIDS.

[60] Naar-King S, Kolmodin K, Parsons JT, Murphy D, ATN 004 Protocol Team, Adolescent Trials Network for HIV/AIDS Interventions (2010). Psychosocial factors and substance use in high-risk youth living with HIV: a multi-site study. AIDS Care.

[61] Balaji AB, Oster AM, Viall AH, Heffelfinger JD, Mena LA, Toledo CA (2012). Role flexing: how community, religion, and family shape the experiences of young black men who have sex with men. AIDS Patient Care STDS.

[62] Lewis LJ, Kertzner RM (2003). Toward improved interpretation and theory building of African American male sexualities. J Sex Res.

[63] Stokes JP, Peterson JL (1998). Homophobia, self-esteem, and risk for HIV among African American men who have sex with men. AIDS Educ Prev.

[64] MacDonell KK, Jacques-Tiura AJ, Naar S, Fernandez MI, ATN 086/106 Protocol Team (2016). Predictors of Self-Reported Adherence to Antiretroviral Medication in a Multisite Study of Ethnic and Racial Minority HIV-Positive Youth. J Pediatr Psychol.

[65] Benjamini Y, Hochberg Y (1995). Controlling the False Discovery Rate - A Practical and Powerful Approach to Multiple Testing. J Royal Stat Soc.

[66] Fitzmaurice GM, Laird NM, Ware JH (2011). Missing Data and Dropout: Overview of Concepts and Methods. Applied Longitudinal Analysis, 2nd Edition.

[67] Patton MQ (1990). Qualitative evaluation and research methods, 2nd edition.

[68] Schutz A, Wagner HR (1970). On Phenomenonology and Social Relations.

[69] Lewis S (2015). Qualitative inquiry and research design: Choosing among five approaches. Health Promot Pract.

[70] Moustakas C (1994). Phenomenological research methods.

[71] Miles MB, Huberman AM (1994). Qualitative Data Analysis: An Expanded Sourcebook.

[72] Yau LHY, Little RJ (2001). Inference for the Complier-Average Causal Effect From Longitudinal Data Subject to Noncompliance and Missing Data, With Application to a Job Training Assessment for the Unemployed. J Am Stat Assoc.

[73] Toh S, Hernán MA (2008). Causal inference from longitudinal studies with baseline randomization. Int J Biostat.

[74] Cohen MS, Chen YQ, McCauley M, Gamble T, Hosseinipour MC, Kumarasamy N, Hakim JG, Kumwenda J, Grinsztejn B, Pilotto JH, Godbole SV, Mehendale S, Chariyalertsak S, Santos BR, Mayer KH, Hoffman IF, Eshleman SH, Piwowar-Manning E, Wang L, Makhema J, Mills LA, de Bruyn G, Sanne I, Eron J, Gallant J, Havlir D, Swindells S, Ribaudo H, Elharrar V, Burns D, Taha TE, Nielsen-Saines K, Celentano D, Essex M, Fleming TR, HPTN 052 Study Team (2011). Prevention of HIV-1 infection with early antiretroviral therapy. N Engl J Med.

[75] Levy A, Johnston K, Annemans L, Tramarin A, Montaner J (2010). The impact of disease stage on direct medical costs of HIV management: a review of the international literature. Pharmacoeconomics.

[76] Lundgren JD, Babiker A, El-Sadr W, Emery S, Grund B, Neaton JD, Neuhaus J, Phillips AN, Strategies for Management of Antiretroviral Therapy (SMART) Study Group (2008). Inferior clinical outcome of the CD4+ cell count-guided antiretroviral treatment interruption strategy in the SMART study: role of CD4+ Cell counts and HIV RNA levels during follow-up. J Infect Dis.

