# Supplementary material for: Epic Allies, a Gamified Mobile Phone App to Improve Engagement in Care, Antiretroviral Uptake, and Adherence Among Young Men Who Have Sex With Men and Young Transgender Women Who Have Sex With Men: Protocol for a Randomized Controlled Trial
Source: JMIR Res Protoc. 2018 Apr 5;7(4):e94. doi: 10.2196/resprot.8811 (PMC5909052; doi:10.2196/resprot.8811)
Supplement: Multimedia Appendix 1 [file resprot_v7i4e94_app1.pdf]

App Features	
**Medication reminders**	Users have the option to set up a maximum of two daily medication reminders through the app; timing can be adjusted at any time (Figure 2). Reminders appear within the app as a pop-up alert (e.g., It's time to take your medication!), and outside of the app as a discreet notification on the user's phone on the lockscreen or as a banner notification (e.g., Your 8:00 PM reminder is going off!).
	Feature relationship to IMB model<br>*Information*: Specifies which medications to take at the appropriate times; *Motivation*: Provides cues to action to increase motivation for behavior change; *Behavioral skills*: Encourages development of new skills to integrate medication routines into daily life.
**User surveys**	All users are initially asked to rate how close they are to starting ART on a scale of 1 to 5 (e.g., 1= not at all ready, 5= on medications). Users not currently on ART are prompted to answer this question once per week. For those on ART, a daily survey appears when the app is opened that compels users to answer a question about daily adherence as well as self-selected behavior tracking questions (alcohol use, marijuana use, exercise, mood, and/or smoking). By default, all behavior questions have tracking turned on; during enrollment, users have the option to accept the default settings, or customize them according to preference. Users receive virtual tokens for all data entry. Tokens are used to play the mini-games available in *Epic Allies*.
	Feature relationship to IMB model<br>Collection of survey data allows for generation of tailored feedback and data visualizations (see Health Center) that address information, motivation, and behavioral skills needs.
**Profile**	The user profile (Figure 3), which can be viewed by other app users, includes the user's chosen alias/superhero name, a customizable avatar (Figure 4), game level, current number of consecutive days taking medications for those on ART or readiness for ART uptake for those not currently on ART, and achievement badges. The following badges can be earned for reaching activity milestones: Perseverance (consecutive days of ART adherence, only available to those on ART), Readiness (begin taking ART) (Figure 5), Allegiance (interact with other participants on the app), Knowledge (read Daily Dose articles and/or successfully complete article quizzes), Strength (complete mini-games), and Awareness (track health behaviors through daily surveys). With the exception of the Readiness badge, which is binary (i.e., yes/no), all badges have rankings from 1 to 9. Higher badge rankings correspond to achievement of more challenging milestones. For example, to achieve rank 1 of the Allegiance badge, users must interact with allies 4 times. To achieve rank 2 of this badge, users must interact with their allies 14 times. Badges provide motivation for users to achieve milestones, give users a sense of progress and accomplishment for their achievements, and allow other users to see these achievements.
	Feature relationship to IMB model<br>*Information:* Badges provide information on adherence and app achievements and areas for improvement; *Motivation:* Visualization of game level and badges motivates users to adhere to medications and engage with the app; *Behavioral skills*: Display of badge achievements increases ART behavior change self-efficacy.
**Health Center**	The Health Center uses survey data to show a calendar view of health progress. This includes a visual representation of adherence for those on ART or a call-to-action for users to begin taking ART for those not currently on ART (Figure 6). Users are also able to see which, if any, behaviors are not being tracked. For the behaviors that are being tracked, each calendar visualization indicates the relationship between days behaviors occurred and days on which ART was missed to encourage participants to consider how these factors (e.g. marijuana use, depressed mood) may be influencing ART adherence. Daily survey data are also used to generate weekly tailored messages based on adherence and other tracked behaviors that are delivered to users through the Health Center (Figure 7).
	Feature relationship to IMB model<br>*Information:* Provide information on ART adherence patterns, including how these patterns are related to other tracked behaviors; *Motivation*: Reinforce positive behaviors and provide motivation for changing negative behaviors; *Behavioral skills*: Positive feedback increases adherence self-

	efficacy.
**Interaction with Allies**	A cornerstone of *Epic Allies* is the interactions between players to foster an environment that supports norms for medication-taking, thereby decreasing stigma related to HIV status and ART use (Figure 8). Players can interact with others app users, known as Allies, in many ways including: 1) sending and receiving encouragement to motivate behavior change; 2) sending and receiving recognition for positive behaviors; and 3) challenging other users to reach behavior goals. The messages are pre-populated (e.g., "You're totally in the zone! You deserve that Awareness medal") to ensure alignment with the needs of the recipient and to maintain participant anonymity.
	Feature relationship to IMB model<br>*Information:* Receive feedback from Allies about adherence and app behaviors; *Motivation*: Messages from Allies provide support and create positive adherence norms; *Behavioral skills*: Peer modeling reinforces positive adherence behaviors.
**Daily Dose**	The Daily Dose "newspaper" includes content designed to improve HIV and ART knowledge, promote disease management and increase motivation and self-efficacy (Figure 9). There are three different formats for providing this information and testing knowledge: 1) users read a brief article and respond to a quiz question based on the article; 2) users read a post with a "Dear Doctor" or "Dear Case Manager" question and answer; and 3) users read role model stories in the Op Ed section that describe a similar youth's experience navigating living with HIV, including engagement in care and ART adherence. Successfully completing an article quiz or reading a Dear Dr. or Op Ed post earns users tokens that can be used to play mini-games. Users who successfully complete a Daily Dose article and/or quiz have access to the article and quiz response for the duration of the day; these users receive a new Daily Dose article the following day. Users who do not successfully complete the Daily Dose article and/or quiz will see that same article until it has been completed.
	Feature relationship to IMB model<br>*Information:* Content provides information to improve HIV and ART knowledge and promotes disease self-management; *Motivation*: Increased knowledge improves motivation for behavior change; *Behavioral skills*: Improvements in knowledge increase ART behavior change self-efficacy.
**Minigames**	*Epic Allies* features 5 minigames, simple games intended to be fun and serve as rewards. With the exception of the Lab minigame, stars are rewarded based on the user's score to help the user gauge his/her performance and encourage the user to improve performance. A user can earn up to 5 stars per session per minigame.<br>• **Lab:** Lab is a "plinko"-style minigame available at level 1+ and serves as a store for users to spend gold coins to earn avatar customization items. To play, the user strategically aims and releases a ball into a bucket that unlocks an avatar customization item (e.g., avatar skin tone, suit color, hero symbol, mask, hairstyle, or hair color).<br>• **Combat Training:** Combat Training is a fast-paced minigame available at level 1+. Users knock down targets, avoid danger zones, and collect gold coins when available. The game ends after the user makes three mistakes (e.g., misses a target or taps a danger zone).<br>• **Rooftop Dash:** Rooftop Dash is an "endless runner" minigame available at level 1+. Users answer a distress signal by racing across city rooftops, jumping over ledges, and collecting gold coins when available. The game ends after the user runs into a building or falls off a ledge.<br>• **Team Mission:** Team Mission is available at level 5+ and encourages users to work together to achieve a common goal (Figure 10). Each level 5+ user can help to defeat a monster over the course of one week using the skills learned by playing the Combat Training minigame. Users can only play once per day and does not require a token. The daily score for each user contributes to the total points needed to collectively defeat the monster. If users successfully defeat the monster at the end of the week, they collect additional gold coins as a prize. Team Mission also features a leaderboard so that users can view the top 5 contributors. This mechanic incentivizes the user to perform well so they

	might also be recognized as a top scorer.<br>• **Mind Mine:** Mind Mine is a memory-challenge minigame that is available at level 7+ (Figure 11). Panels on a bomb temporarily flip over to reveal a series of symbols. The user must tap out the symbols in the correct order to successfully diffuse the bomb. Users are also able to collect gold coins when available. The game ends after the user makes three mistakes.
	Feature relationship to IMB model<br>*Motivation:* Minigames are increase motivation to engage with the app and change behaviors.
